# Supplementary material for: Transcription Factor CmNAC34 Regulated CmLCYB-Mediated β-Carotene Accumulation during Oriental Melon Fruit Ripening
Source: Int J Mol Sci. 2022 Aug 29;23(17):9805. doi: 10.3390/ijms23179805 (PMC9455964; doi:10.3390/ijms23179805)
Supplement: Supplementary file 1 [file ijms-23-09805-s001.zip › ijms-1861101-supplementary.pdf]

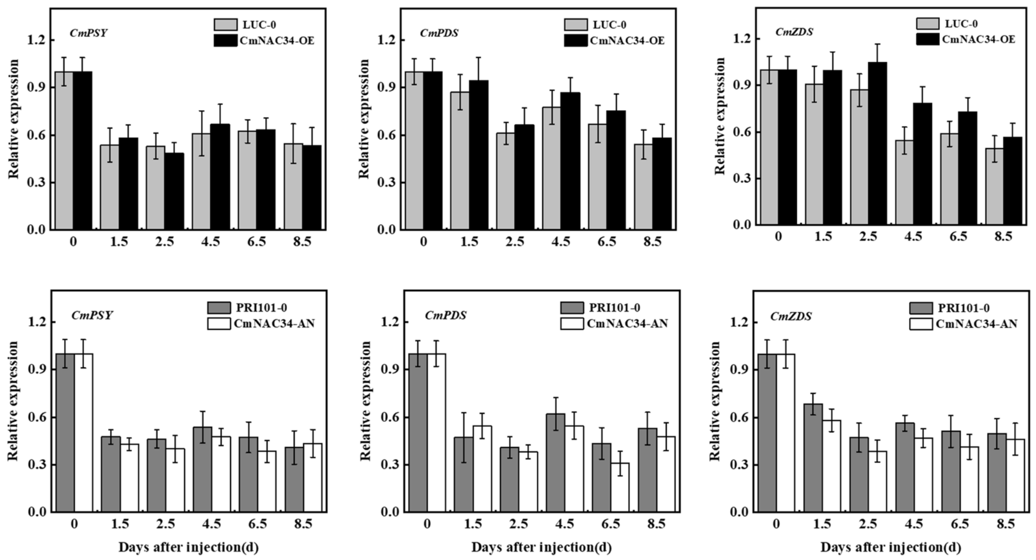

Supplemental Figure S1. The relative expression of carotenoid synthetic genes in transiently infiltrated fruit at different days after injection. Three biological replicates were performed, and the error bar represents the *SE*.
